# LncRNA TNK2‐AS1 regulated ox‐LDL‐stimulated HASMC proliferation and migration via modulating VEGFA and FGF1 expression by sponging miR‐150‐5p

**DOI:** 10.1111/jcmm.14575

**Published:** 2019-08-29

**Authors:** Tianzhi Cai, Xiuzhen Cui, Kelin Zhang, Anji Zhang, Baixue Liu, Jian‐jun Mu

**Affiliations:** ^1^ Department of Cardiology the First Affiliated Hospital of Xi'an Jiaotong University Xi'an China; ^2^ Department of Cardiology the First Affiliated Hospital of Xi'an Medical University Xi'an China; ^3^ Department of Ophthalmology the First Affiliated Hospital of Xi'an Medical University Xi'an China

**Keywords:** fibroblast growth factor 1, human aortic smooth muscle cells, miR‐150‐5p, proliferation and migration, TNK2‐AS1, vascular endothelial growth factor A

## Abstract

Long non‐coding RNAs (lncRNAs) have been indicated for the regulatory roles in cardiovascular diseases. This study determined the expression of lncRNA TNK2 antisense RNA 1 (TNK2‐AS1) in oxidized low‐density lipoprotein (ox‐LDL)‐stimulated human aortic smooth muscle cells (HASMCs) and examined the mechanistic role of TNK2‐AS1 in the proliferation and migration of HASMCs. Our results demonstrated that ox‐LDL promoted HASMC proliferation and migration, and the enhanced proliferation and migration in ox‐LDL‐treated HASMCs were accompanied by the up‐regulation of TNK2‐AS1. In vitro functional studies showed that TNK2‐AS1 knockdown suppressed cell proliferation and migration of ox‐LDL‐stimulated HASMCs, while TNK2‐AS1 overexpression enhanced HASMC proliferation and migration. Additionally, TNK2‐AS1 inversely regulated miR‐150‐5p expression via acting as a competing endogenous RNA (ceRNA), and the enhanced effects of TNK2‐AS1 overexpression on HASMC proliferation and migration were attenuated by miR‐150‐5p overexpression. Moreover, miR‐150‐5p could target the 3’ untranslated regions of vascular endothelial growth factor A (VEGFA) and fibroblast growth factor 1 (FGF1) to regulate FGF1 and VEGFA expression in HASMCs, and the inhibitory effects of miR‐150‐5p overexpression in ox‐LDL‐stimulated HASMCs were attenuated by enforced expression of VEGFA and FGF1. Enforced expression of VEGFA and FGF1 also partially restored the suppressed cell proliferation and migration induced by TNK2‐AS1 knockdown in ox‐LDL‐stimulated HASMCs, while the enhanced effects of TNK2‐AS1 overexpression on HASMC proliferation and migration were attenuated by the knockdown of VEGFA and FGF1. Collectively, our findings showed that TNK2‐AS1 exerted its action in ox‐LDL‐stimulated HASMCs via regulating VEGFA and FGF1 expression by acting as a ceRNA for miR‐150‐5p.

## 1 INTRODUCTION

Atherosclerosis is a vascular disorder characterized by the accumulation of fibrous elements and lipids in the vascular wall and is a major cause of cardiovascular disease‐related deaths.^1^ In China, the incidence of atherosclerosis is expected to increase annually, which imposed substantial medical and economic burden on the society.^2^, ^3^ To date, the pathophysiology of atherosclerosis remains unclear, and recent evidence pointed to vascular smooth muscle cells (VSMCs) as the key factor in the development of atherosclerosis.^4^, ^5^ Studies showed that abnormal VSMC proliferation, migration and synthesis of extracellular matrix by VSMCs contribute to the formation of the atherosclerotic plaque.^6^, ^7^ Unfortunately, the molecular mechanisms that control VSMC proliferation and migration were understudied. Thus, identification of novel targets that can restore the abnormal cellular functions of VSMCs may have therapeutic potentials for the atherosclerosis.

Studies from human genome projects showed that more than 90% of the transcripts in human genome were incapable of coding proteins,^8^ and among these non‐coding RNA, the long non‐coding RNAs (lncRNAs) have more than 200 nucleotides in length^9^ and have diverse biology functions such as cell proliferation, cell apoptosis, cell migration and cell metabolism.^9^ Various diseases including cancer, cardiovascular diseases and neurodegenerative diseases were found to involve in the dysregulated lncRNAs.^10^ In the aspect of atherosclerosis, lncRNA retinal non‐coding RNA 3 knockdown promoted atherosclerosis by enhancing endothelial cell and VSMC proliferation.^11^ The lncRNA UCA1 was up‐regulated in oxidized low‐density lipoprotein (ox‐LDL)‐stimulated VSMCs and promoted VSMC migration and proliferation via suppressing miR‐26a expression,^12^ which contributed to the development of atherosclerosis. In addition, Huang et al showed that lncRNA H19 regulated the atherosclerosis via increasing acid phosphatase 5 expression.^13^ Recently, the lncRNA TNK2 antisense RNA 1 (TNK2‐AS1) was found to be up‐regulated in non‐small cell lung cancer tissues and TNK2‐AS1 could promote the angiogenesis of non‐small cell lung cancer via STAT3/vascular endothelial growth factor A (VEGFA) signalling.^14^ However, the role of TNK2‐AS1 in the cellular functions of VSMCs has not been explored yet.

In this study, we determined the expression of TNK2‐AS1 in ox‐LDL‐stimulated human aortic smooth muscle cells (HASMCs) and examined the effects of TNK2‐AS1 on the proliferation and migration of HASMCs. Additionally, the underlying regulatory pathways of TNK2‐AS1 were also explored by in vitro assays. The present study may provide us with better understanding regarding the role of lncRNA in HASMC proliferation and migration.

## 2 MATERIALS AND METHODS

### 2.1 Cell lines and cell culture and ox‐LDL treatment

The HASMCs were purchased from ScienCell (Carlsbad, USA). The HASMCs are grown in the smooth muscle cell growth medium supplied with 10% foetal bovine serum (FBS; Thermo Fisher Scientific, Waltham, USA) and antibiotics (100 U/mL penicillin/streptomycin). The HASMCs were maintained at 37°C in a humidified cell incubation chamber with 5% CO_2_. The ox‐LDL was a commercial product from Sigma‐Aldrich (St. Louis, USA) and the treatment concentrations for ox‐LDL were 25, 50 and 100 µg/mL, and treatment duration was 24 and 48 hours according to different experimental set‐ups.

### 2.2 Small interfering RNAs (siRNAs), plasmid constructs, microRNA (miRNA) oligonucleotides and in vitro transfections

The siRNAs for TNK2‐AS1 (named as si‐TNK2‐AS1 (#1) and si‐TNK2‐AS1 (#2)), VEGFA, fibroblast growth factor 1 (FGF1) and scrambled negative control siRNAs (si‐NC) were designed and synthesized by RiboBio (Guangzhou, China). The pcDNA3.1 plasmid was used to generate VEGFA‐ and FGF1‐overexpressing vector (named as pcDNA3.1‐VEGFA and pcDNA3.1‐FGF1), and these constructs were obtained from GenePharma (Shanghai, China). The miRNA oligonucleotides including miR‐150‐5p mimic, miR‐150‐5p inhibitor as well as the respective negative controls were purchased from Ambion (Waltham, USA). The in vitro transfections were done by using the Lipofectamine 2000 reagent (Invitrogen, Carlsbad, USA), and transfected cells were subjected to different in vitro assays at 24 hours after transfection.

### 2.3 Quantitative real‐time polymerase chain reaction (qRT‐PCR)

Total RNA from treated HASMCs was isolated using TRIzol reagent (Invitrogen). For the quantification of lncRNA and mRNA, the cDNA was obtained by using PrimeScript RT reagent kit (Takara, Dalian, China). The real‐time PCR was performed on the 7900 Real‐time PCR System using SYBR Premix Ex Taq (Takara). For the quantification of miR‐150‐5p, cDNA was obtained by using Taqman MicroRNA Reverse Transcription kit (Thermo Fisher Scientific, Waltham, USA) and real‐time PCR was performed on the 7900 Real‐time PCR System using Taqman microRNA assay kit (Thermo Fisher Scientific). The fold changes for lncRNA, mRNA and miRNA expression were determined using comparative Ct method, and GAPDH was selected as the internal control for lncRNA and mRNA and U6 was selected as the internal control for miRNA.

### 2.4 Cell Counting Kit‐8 (CCK‐8) assay

CCK‐8 (Dojindo Laboratories, Kumamoto, Japan) was used to determine cell proliferative ability of HASMCs. Briefly, the treated HASMCs were seeded onto 96‐well plates at a density of 1x10^4^ cells/well with a volume of 100 µL. At the indicated time, the HASMCs were incubated with 10 µL CCK‐8 solution for 30 minutes at 37°C. After that, the cell proliferative index was determined by measuring the absorbance at 450 nm wavelength.

### 2.5 Transwell migration assay

The migratory potential of HASMCs was evaluated by the Transwell migration assay. Briefly, the treated HASMCs (5 × 10^4^ cells) were pre‐incubated with mitomycin C (20 μmol/L) for 2 hours and were re‐suspend in the smooth muscle cell growth medium (300 µL) without FBS and were seeded on the upper Transwell chamber with inserts (8 µm pore size; Corning, USA). The lower chamber was filled with culture medium with 10% FBS as the chemo‐attractant. After a further incubation for 24 hours, HASMCs on the top surface of the membrane were cleaned with a cotton swab, and the HASMCs migrated into the lower membrane were fixed with 4% paraformaldehyde for 15 minutes and were then stained with 0.1% crystal violet for 10 minutes at room temperature. Cell migratory potential was evaluated by counting the stained cells using a light microscope by randomly selecting five fields.

### 2.6 Luciferase reporter assay

The fragments of TNK2‐AS1 and the 3’ untranslated region (3’UTR) of VEGFA and FGF1 were amplified by PCR and were then subcloned into the pGL3 reporter vector (Promega, Madison, USA). For the corresponding mutant vectors, the mutated sites were generated by using site‐directed mutagenesis kit (Agilent, Santa Clara, USA), and then the mutated segments were also subcloned into the pGL3 reporter vector. For the luciferase reporter assay, HASMCs were seeded on the 24‐well plates at the 70%‐80% confluence. After overnight incubation, HASMCs were cotransfected with miRNAs and pGL3 reporter constructs using the Lipofectamine 2000 reagent, according to the manufacturer's protocol. At 48 hours after transfection, the luciferase activity in HASMCs was determined by the Dual‐Luciferase Reporter Assay kit (Promega). The relative luciferase activity was calculated by the ratio between Firefly luciferase activity and the Renilla luciferase activity.

### 2.7 Western blot assay

Total proteins from cells were extracted on the ice‐cold RIPA lysis buffer with protease inhibitors (Sigma, St. Louis, USA). The concentrations of the extracted proteins were determined by the BCA assay kit (Beyotime, Beijing, China). Equal amounts of extracted proteins were separated by electrophoresis on the 10% sodium dodecyl sulphate‐polyacrylamide gel and were then transferred onto the polyvinylidene difluoride membranes (Sigma). The transferred membranes were then blocked with 5% non‐fat milk in Tris‐buffered saline with Tween‐20 (TBST). The membranes were then probed by using primary antibodies against proliferating cell nuclear antigen (PCNA), α‐smooth muscle action (α‐SMA) and β‐actin at 4°C overnight. After being washed with TBST for 5 minutes × 3 times, the membranes were probed by using horseradish peroxidase‐conjugated secondary antibody at room temperature for 2 hours. The Western blot bands were visualized by ECL kit (Thermo Fisher Scientific). β‐Actin was used as the internal for targeted protein expression.

### 2.8 Statistical analysis

The data were analysed, and the graphs were plotted by the GraphPad Prism Version 6.0 (GraphPad Software Company, La Jolla, USA). The data were presented as mean ± standard deviation (SD) of at least three determinations. The significance for the differences between different treatment groups was assessed by the unpaired Student's *t* test or one‐way ANOVA followed by Bonferroni's post hoc test. Statistical significance was considered when *P* < .05.

## 3 RESULTS

### 3.1 Ox‐LDL promoted cell proliferation and up‐regulated TNK2‐AS1 expression in HASMCs

**Figure 1:**
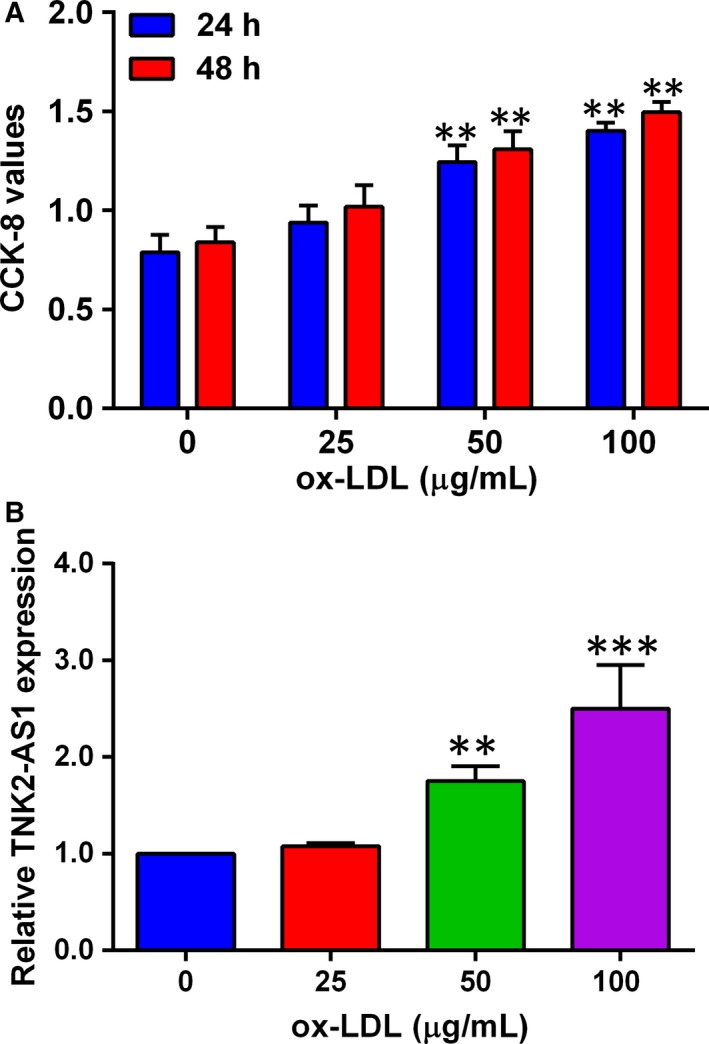
ox‐LDL promoted cell proliferation and TNK2‐AS1 expression in HASMCs. (A) The HASMCs were treated with different concentrations of ox‐LDL for 24 or 48 h, and cell proliferation was determined by CCK‐8 assay. (B) The HASMCs were treated with ox‐LDL for 24 h, and TNK2‐AS1 expression was determined by qRT‐PCR assay. N = 3. Significant differences between treatment groups and control group were shown as **P < .01 and ***P < .001

As previous studies have demonstrated that ox‐LDL promoted VSMC proliferation,^15^ we confirmed the effects of ox‐LDL on the proliferation of HASMCs. As shown in Figure 1A, HASMCs were treated with ox‐LDL at the concentration ranges from 25 µg/mL to 100 µg/mL for 24 and 48 hours, respectively, and ox‐LDL treatment significantly potentiated HASMC proliferation in a concentration‐ and time‐dependent manner (Figure 1A). TNK2‐AS1 expression was also examined in HASMCs after being treated with ox‐LDL (25‐100 µg/mL) for 24 hours, and ox‐LDL at 50 µg/mL and 100 µg/mL significantly up‐regulated TNK2‐AS1 expression in HASMCs as determined by qRT‐PCR (Figure 1B).

### 3.2 Knockdown of TNK2‐AS1 attenuated the hyper‐proliferation and migration induced by ox‐LDL in HASMCs

**Figure 2:**
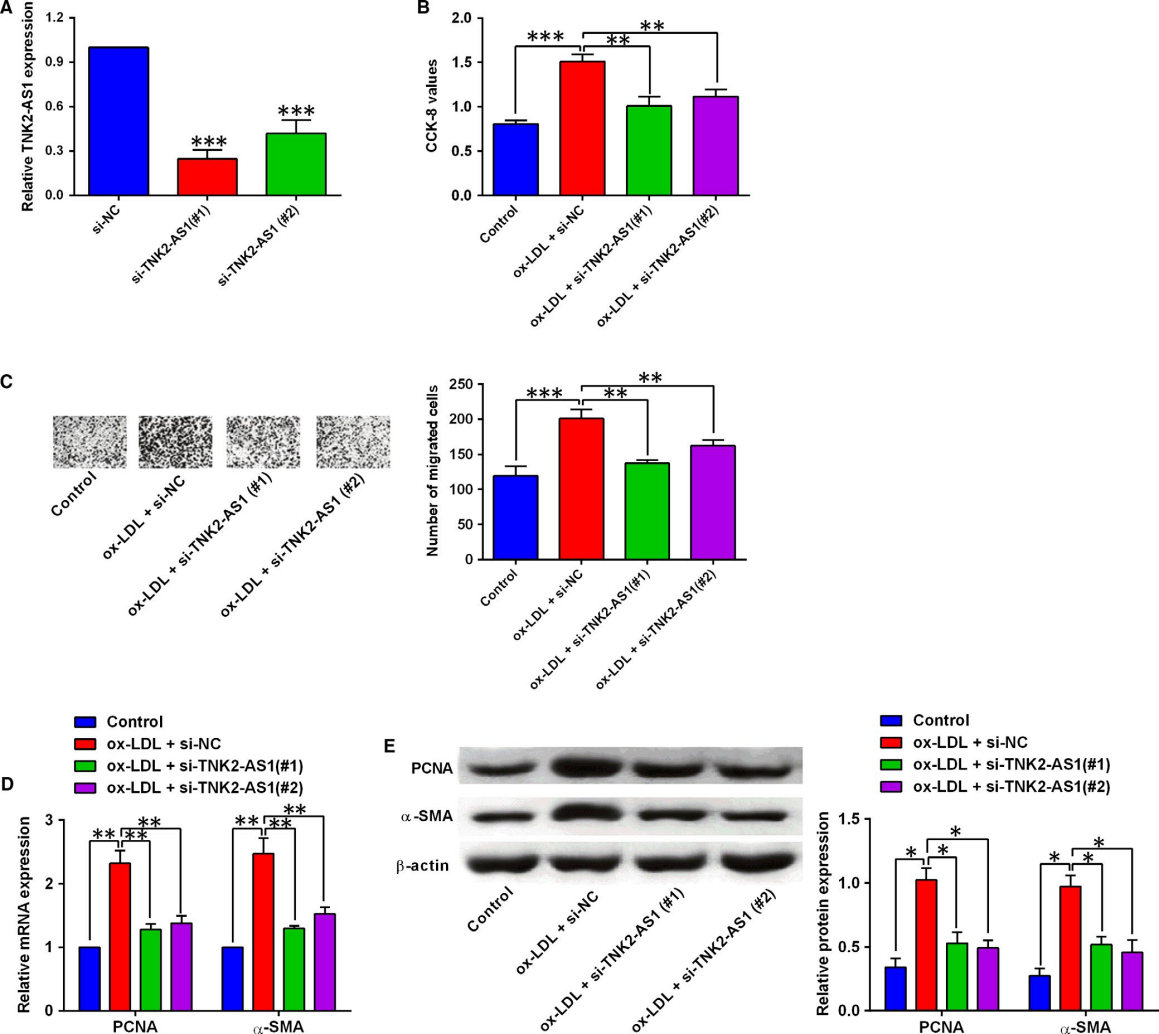
Knockdown of TNK2‐AS1 suppressed cell proliferation and migration of ox‐LDL‐stimulated HASMCs. (A) The HASMCs were transfected with scrambled siRNA (si‐NC) or TNK2‐AS1 siRNAs (siTNK2‐AS1(#1) and (#2)), and at 24 h after transfection, TNK2‐AS1 expression was determined by qRT‐PCR. (B‐E) The HASMCs were pre‐transfected with si‐NC, si‐TNK2‐AS1(#1) or (#2), and 24 h later, HASMCs were treated with ox‐LDL (100 µg/mL) for 24 h, and cell proliferation was determined by CCK‐8 assay (B), cell migration was determined by Transwell migration assay (C), the expression of PCNA and α‐SMA was determined by qRT‐PCR (D) and Western blot assay (E). N = 3. Significant differences between treatment groups and control group were shown as *P < .05, **P < .01 and ***P < .001

Knockdown of TNK2‐AS1 in HASMCs was confirmed by qRT‐PCR after cells being transfected with TNK2‐AS1 siRNAs, and transfection with TNK2‐AS1 siRNAs, that is, si‐TNK2‐AS1 (#1) and (#2) both significantly suppressed TNK2‐AS1 expression in HASMCs (Figure 2A). The CCK‐8 assay and Transwell migration assay showed that ox‐LDL treatment promoted HASMC proliferation and migration, and the transfection with TNK2‐AS1 siRNAs significantly reversed the ox‐LDL‐mediated effects on HASMC proliferation and migration (Figure 2B and 2). Further qRT‐PCR and Western blot assays were performed to determine the expression of PCNA and α‐SMA in HASMCs, and 100 µg/mL ox‐LDL treatment for 24 hours up‐regulated the expression of PNCA and α‐SMA, which was attenuated by TNK2‐AS1 siRNAs (si‐TNK2‐AS1 (#1) and (#2)) transfection in HASMCs (Figure 2D and 2).

### 3.3 Overexpression of TNK2‐AS1 promoted cell proliferation and migration of HASMCs

**Figure 3:**
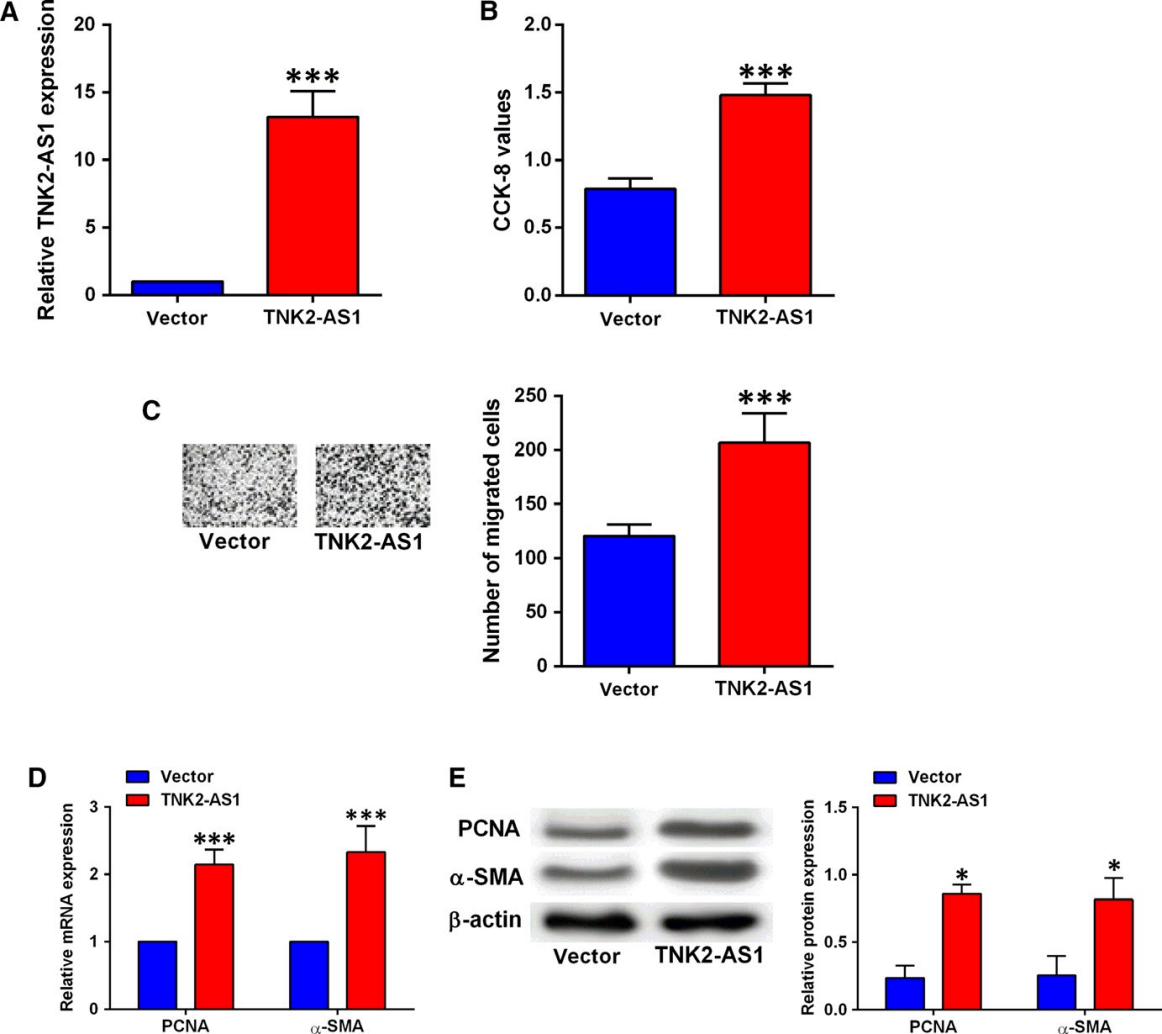
Overexpression of TNK2‐AS1 promoted cell proliferation and migration of HASMCs. (A) The HASMCs were transfected with pcDNA3.1 (vector) or pcDNA3.1‐TNK2‐AS1, and at 24 h after transfection, TNK2‐AS1 expression was determined by qRT‐PCR. (B‐E) The HASMCs were transfected with pcDNA3.1 or pcDNA3.1‐TNK2‐AS1, and 24 h later, cell proliferation was determined by CCK‐8 assay (B), cell migration was determined by Transwell migration assay (C), the expression of PCNA and α‐SMA was determined by qRT‐PCR (D) and Western blot assay (E). N = 3. Significant differences between treatment groups and control group were shown as *P < .05, ***P < .001

Overexpression of TNK2‐AS1 in HASMCs was confirmed by qRT‐PCR after cells being transfected with pcDNA3.1 or pcDNA3.1‐TNK2‐AS1, and transfection with pcDNA3.1‐TNK2‐AS1 significantly up‐regulated TNK2‐AS1 expression in HASMCs (Figure 3A). The CCK‐8 and Transwell migration assays showed that TNK2‐AS1 overexpression promoted HASMC proliferation and migration (Figure 3B and 3). Consistently, qRT‐PCR and Western blot results showed that TNK2‐AS1 overexpression increased the mRNA and protein expression levels of both PNCA and α‐SMA in HASMCs (Figure 3D and 3). In addition, the treatment with ox‐LDL caused a marginal increase (not statistically significant) in cell proliferation and migration of HASMCs with TNK2‐AS1 overexpression (Figure S1).

### 3.4 TNK2‐AS1 regulated cell proliferation and migration via sponging miR‐150‐5p in HASMCs

**Figure 4:**
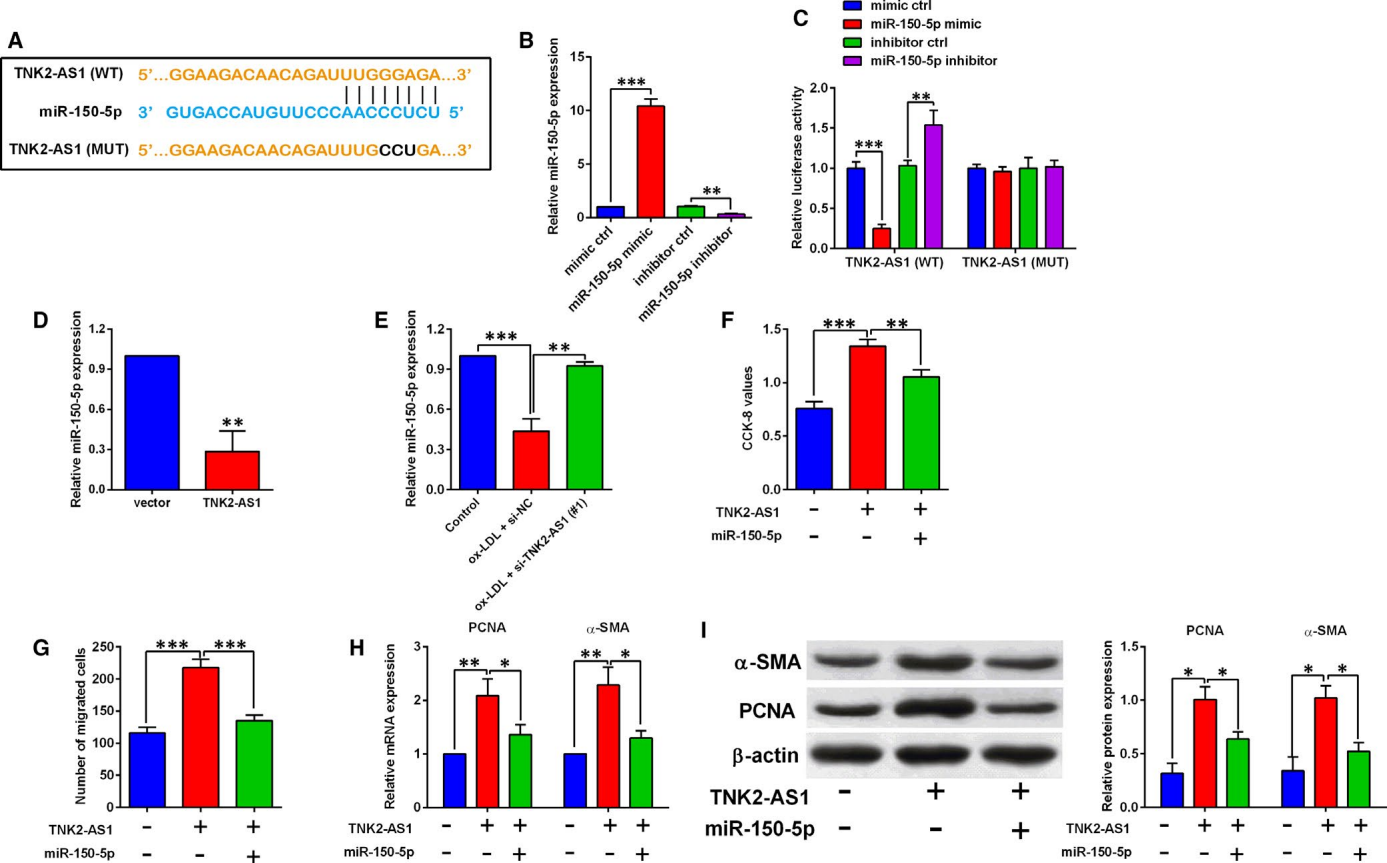
TNK2‐AS1 regulated cell proliferation and migration via sponging miR‐105‐5p in HASMCs. (A) The predicted binding sequence between TNK2‐AS1 and miR‐150‐5p was analysed by LncBase Predicted V2.0. (B) The HASMCs were transfected with mimic ctrl, miR‐150‐5p mimic, inhibitor ctrl or miR‐105‐5p inhibitor, and at 24 h after transfection, miR‐150‐5p expression was determined by qRT‐PCR. (C) The HASMCs were cotransfected with miRNAs and reporter vectors, and at 48 h after cotransfection, the luciferase activity in HASMCs was determined by dual‐luciferase reporter assay. (D) The HASMCs were transfected with pcDNA3.1 or pcDNA3.1‐TNK2‐AS1, and at 24 h after transfection, miR‐150‐5p expression was determined by qRT‐PCR. (E) The HASMCs were pre‐transfected with si‐NC or TNK2‐AS1 siRNA, and 24 h later, cells were treated with ox‐LDL (100 µg/mL) for 24 h, and miR‐150‐5p expression was determined by qRT‐PCR. (F‐I) The HASMCs were cotransfected with pcDNA3.1 (−) + mimic NC (−), pcDNA3.1‐TNK2‐AS1 (+) + mimic NC (−), or pcDNA3.1‐TNK2‐AS1 (+) + miR‐150‐5p mimic (+), and at 24 h after transfection, cell proliferation was determined by CCK‐8 assay (F), cell migration was determined by Transwell migration assay (G), the expression of PCNA and α‐SMA was determined by qRT‐PCR (H) and Western blot assay (I). N = 3. Significant differences between treatment groups and control group were shown as *P < .05, **P < .01 and ***P < .001

The downstream miRNA that could be potentially targeted by TNK2‐AS1 was predicted by LncBase Predicted V2.0 online software, and miR‐150‐5p was selected for further examination in our study. Treatment with miR‐150‐5p mimic markedly increased the miR‐150‐5p expression by around 10‐fold when compared to mimic ctrl group (Figure 4B), while treatment with miR‐150‐5p inhibitor suppressed miR‐150‐5p expression in HASMCs (Figure 4B). The interaction between miR‐150‐5p and TNK2‐AS1 was further confirmed by luciferase reporter assay, and we constructed the reporter vectors containing the wild‐type or mutant fragments of TNK2‐AS1 (Figure 4A), and different miRNAs and reporter vectors were cotransfected into HASMCs, and at 48 hours after transfection, miR‐150‐5p overexpression inhibited the luciferase activity of the wild‐type reporter vector, but not the mutant one (Figure 4C), while miR‐150‐5p knockdown increased the luciferase activity of the wild‐type reporter vector, but not the mutant one (Figure 4C). As expected, TNK2‐AS1 overexpression suppressed miR‐150‐5p expression in HASMCs (Figure 4D), and ox‐LDL (100 µg/mL) treatment for 24 hours also down‐regulated miR‐150‐5p expression in HASMCs, which was attenuated by TNK2‐AS1 siRNA transfection (Figure 4E). Further rescue experiments showed that TNK2‐AS1 overexpression promoted cell proliferation and migration, and up‐regulated PCNA and α‐SMA expression in HASMCs, which was partially reversely by the presence of miR‐150‐5p mimic (Figure 4F‐I).

### 3.5 MiR‐150‐5p regulated cell proliferation and migration of HASMCs via targeting VEGFA and FGF1

**Figure 5:**
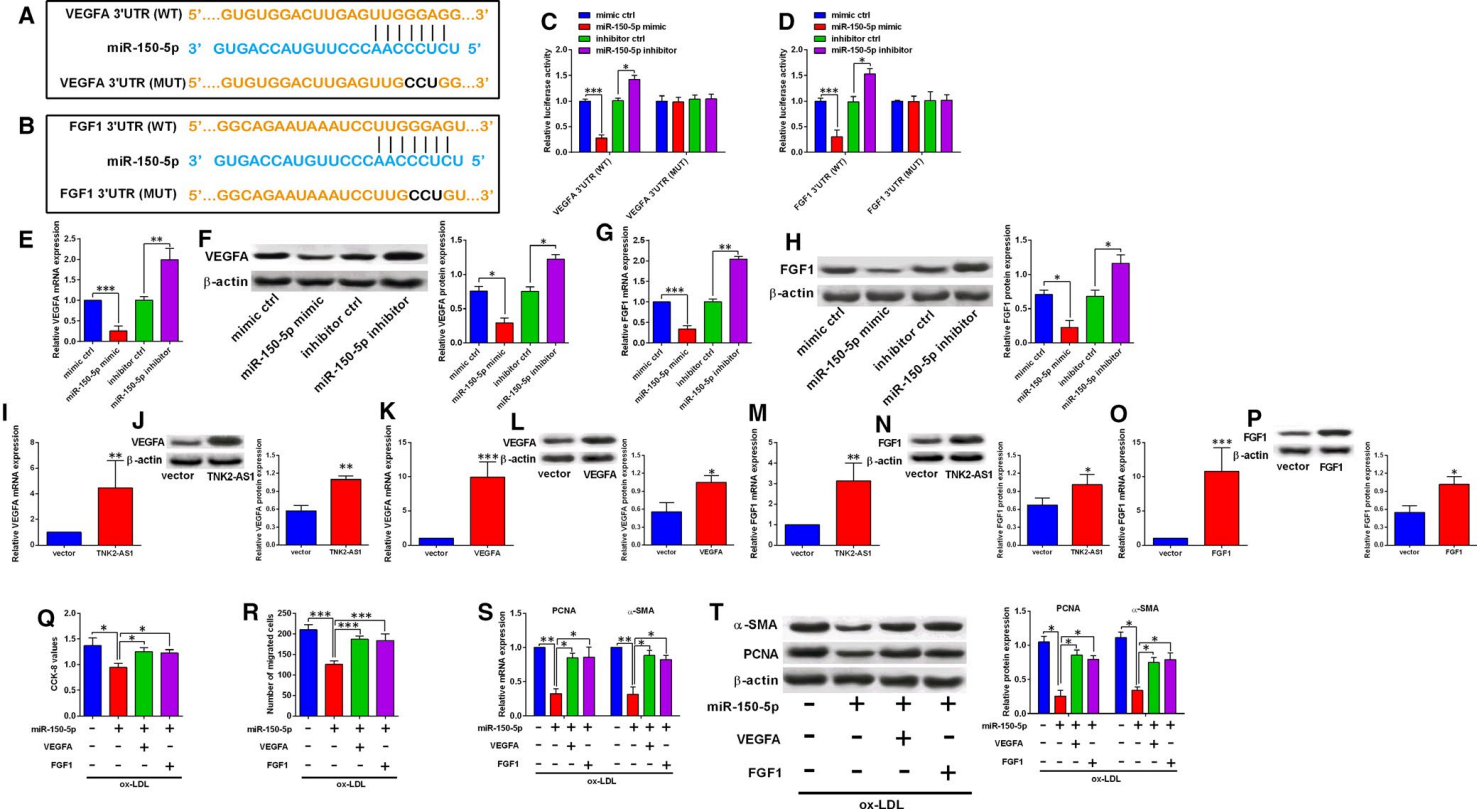
MiR‐150‐5p regulated cell proliferation and migration of HASMCs via targeting VEGFA and FGF1. (A‐B) The predicted binding sequence between miR‐150‐5p and VEGFA 3’UTR (A) or FGF1 3’UTR (B) was analysed by TargetScan. (C‐D) The HASMCs were cotransfected with miRNAs and reporter vectors containing wild‐type/mutant VEGFA 3’UTR (C) or FGF1 3’UTR (D), and at 48 h after cotransfection, the luciferase activity in HASMCs was determined by dual‐luciferase reporter assay. (E‐H). The HASMCs were transfected with different miRNAs, and at 24 h after transfection, the mRNA and protein expression levels of VEGFA (E‐F) and the mRNA and protein expression levels of FGF1 were determined by qRT‐PCR and Western blot assays. (I‐J) The HASMCs were transfected with pcNDA3.1 or pcDNA3.1‐TNK2‐AS1, and 24 h after transfection, the mRNA and protein expression levels of VEGFA were determined by qRT‐PCR and Western blot assay. (K‐L) The HASMCs were transfected with pcNDA3.1 or pcDNA3.1‐VEGFA, and 24 h after transfection, the mRNA and protein expression levels of VEGFA were determined by qRT‐PCR and Western blot assay. (M‐N) The HASMCs were transfected with pcNDA3.1 or pcDNA3.1‐TNK2‐AS1, and 24 h after transfection, the mRNA and protein expression levels of FGF1 were determined by qRT‐PCR and Western blot assay. (O‐P) The HASMCs were transfected with pcNDA3.1 or pcDNA3.1‐FGF1, and 24 h after transfection, the mRNA and protein expression levels of FGF1 were determined by qRT‐PCR and Western blot assay. (Q‐T) The HASMCs were first cotransfected with different miRNAs and plasmids, and 24 h later, cells were treated with ox‐LDL (100 µg/mL) for 24 h, cell proliferation was determined by CCK‐8 assay (Q), cell migration was determined by Transwell migration assay (R), the expression of PCNA and α‐SMA was determined by qRT‐PCR (S) and Western blot assay (T). N = 3. Significant differences between treatment groups and control group were shown as *P < .05, **P < .01 and ***P < .001

To further understand the involvement of miR‐150‐5p in HASMC proliferation and migration, we identified VEGFA and FGF1 as potential targets of miR‐150‐5p using TargetScan software (Figure 5A‐B). To confirm the interaction between miR‐150‐5p and the 3’UTRs of VEGFA and FGF1, we further constructed the reporter vectors containing wild‐type/mutant VEGFA 3’UTR or FGF1 3’UTR. MiR‐150‐5p overexpression inhibited the luciferase activity of reporter vector with wild‐type VEGFA 3’UTR, but not the mutant one (Figure 5C), while miR‐150‐5p knockdown increased the luciferase activity of the reporter vector with wild‐type VEGFA 3’UTR, but not the mutant one (Figure 5C). Similar findings were observed for reporter vectors containing wild‐type/mutant FGF1 3’UTR (Figure 5D). The qRT‐PCR and Western blot assays showed that miR‐150‐5p overexpression suppressed both VEGFA and FGF1 expression (Figure 5E‐H), while miR‐150‐5p knockdown increased both VEGFA and FGF1 expression in HASMCs (Figure 5E‐H). In addition, transfection with pcDNA3.1‐TNK2‐AS1 or pcDNA3.1‐VEGFA up‐regulated VEGFA expression in HASMCs (Figure 5I‐L). Similarly, transfection with pcDNA3.1‐TNK2‐AS1 or pcDNA3.1‐FGF1 also up‐regulated FGF1 expression in HASMCs (Figure 5M‐P). The in vitro rescue assays further showed that miR‐150‐5p overexpression suppressed cell proliferation and migration, and also down‐regulated PCNA and α‐SMA expression in HASMCs, while VEGFA overexpression and FGF1 overexpression both significantly attenuated the inhibitory actions of miR‐150‐5p mimic transfection in HASMCs (Figure 5Q‐T).

### 3.6 TNK2‐AS1 regulated cell proliferation and migration via targeting VEGFA and FGF1 in ox‐LDL‐treated HASMCs

**Figure 6:**
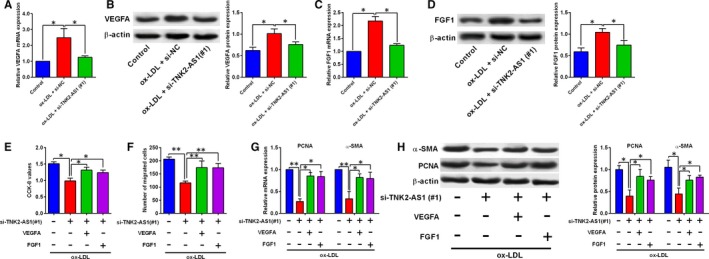
TNK2‐AS1 regulated cell proliferation and migration via targeting VEGFA and FGF1 in ox‐LDL‐treated HASMCs. (A‐D) The HASMCs were transfected with si‐NC or si‐TNK2‐AS1(#1), and at 24 h after transfection, cells were treated with ox‐LDL (100 µg/mL) for 24 h, the mRNA and protein expression levels of VEGFA (A‐B), the mRNA and protein expression levels of FGF1 (C‐D) were determined by qRT‐PCR and Western blot assay. (E‐F) The HASMCs were first cotransfected with different siRNAs and plasmids, and 24 h later, cells were treated with ox‐LDL (100 µg/mL) for 24 h, cell proliferation was determined by CCK‐8 assay (E), cell migration was determined by Transwell migration assay (F), the expression of PCNA and α‐SMA was determined by qRT‐PCR (G) and Western blot assay (H). N = 3. Significant differences between treatment groups and control group were shown as *P < .05, **P < .01

Furthermore, ox‐LDL (100 µg/mL) up‐regulated VEGFA expression in HASMCs, and transfection with TNK2‐AS1 siRNA down‐regulated VEGFA expression in ox‐LDL‐treated HASMCs (Figure 6A‐B). Similar findings were also found for the expression of FGF1 (Figure 6C‐D). The rescue experiments showed that knockdown of TNK2‐AS1 decreased cell proliferative and migratory abilities, and also suppressed the PCNA and α‐SMA expression in ox‐LDL‐treated HASMCs (Figure 6E‐H). The transfection with pcDNA3.1‐VEGFA and the transfection with FGF1 both attenuated the inhibitory effects of TNK2‐AS1 knockdown on cell proliferation and migration of ox‐LDL‐treated HASMCs (Figure 6E‐H). On the other hand, transection with VEGFA siRNA and FGF1 siRNA, respectively, suppressed the VEGFA and FGF1 expression in HASMCs (Figure S2a‐d). In vitro functional assays showed that the enhanced effects of TNK2‐AS1 overexpression on HASMC proliferation and migration were attenuated by the knockdown of VEGFA and FGF1 (Figure S2e‐f).

## 4 DISCUSSION

Various studies have demonstrated that hyper‐proliferation and enhanced migration of VSMCs contributed to the pathogenesis of atherosclerosis,^16^ and finding novel targets that repress VSMC proliferation and migration may represent an effective strategy for alleviating atherosclerosis. In the present study, we found that ox‐LDL, a well‐documented risk contributor for atherosclerosis,^15^ promoted HASMC proliferation and migration, and the enhanced proliferation and migration in ox‐LDL‐treated HASMCs was accompanied by the up‐regulation of TNK2‐AS1. In vitro functional studies showed that TNK2‐AS1 knockdown suppressed cell proliferation and migration of ox‐LDL‐stimulated HASMCs, while TNK2‐AS1 overexpression enhanced HASMC proliferation and migration. Additionally, TNK2‐AS1 was found to inversely regulate miR‐150‐5p expression via acting as a competing endogenous RNA (ceRNA), and the enhanced effects of TNK2‐AS1 overexpression on HASMC proliferation and migration were attenuated by miR‐150‐5p overexpression. Moreover, miR‐150‐5p could target the 3’UTRs of VEGFA and FGF1 to regulate VEGFA and FGF1 expression in HASMCs, and the inhibitory effects of miR‐150‐5p overexpression in ox‐LDL‐stimulated HASMCs were attenuated by enforced expression of VEGFA and FGF1. Consistently, enforced expression of VEGFA and FGF1 also partially restored the suppressed cell proliferation and migration induced by TNK2‐AS1 knockdown in ox‐LDL‐stimulated HASMCs; while knockdown of VEGFA and FGF1 both significantly attenuated the enhanced effects of TNK2‐AS1 up‐regulation on HASMC proliferation and migration. Collectively, our findings showed the potential role of TNK2‐AS1 in regulating cell proliferation and migration in the ox‐LDL‐stimulated cellular model of atherosclerosis.

The role of lncRNA in regulating cell proliferation and migration in ox‐LDL‐stimulated cellular model of atherosclerosis has been reported in various studies so far. The lncRNA HOXC antisense RNA 1 suppressed the accumulation of cholesterol induced by ox‐LDL in macrophages via up‐regulating homeobox C6 expression.^17^ The lncRNA growth arrest specific 5 from exosomes was increased in ox‐LDL‐stimulated endothelial cells and in atherosclerotic tissues from patients and promoted endothelial cell apoptosis.^18^ The lncRNA myocardial infarction associated transcript (MIAT) was up‐regulated in the serum of the atherosclerotic patients and the ox‐LDL‐treated HASMCs, and lncRNA MIAT overexpression promoted HASMCs proliferation and hindered cell apoptosis via modulating miR‐181b/STAT3 axis.^19^ Additionally, lncRNA H19 was increased in the serum of atherosclerotic patients and the ox‐LDL‐stimulated HASMCs, and H19 knockdown inhibited cell proliferation and induced cell apoptosis in ox‐LDL‐stimulated HASMCs via miR‐148/β‐catenin axis.^20^ Recently, the lncRNA maternally expressed 8 (MEG8) was identified to be down‐regulated in ox‐LDL‐stimulated VSMCs and enhanced MEG8 expression suppressed VSMC proliferation and migration via regulating miR‐181a/PPARα signalling pathways.^21^ In our data, we showed that the up‐regulation of TNK2‐AS1 in the ox‐LDL‐stimulated HASMCs and enhanced TNK2‐AS1 expression promoted HASMC proliferation and migration, while TNK2‐AS1 knockdown inhibited ox‐LDL‐stimulated HASMC proliferation and migration, suggesting that TNK2‐AS1 exerts enhanced effects on HASMC proliferation and migration.

To further elaborate the role of TNK2‐AS1 in HASMC proliferation and migration, we examined if TNK2‐AS1 could act as ceRNA. LncBase Predicted V2.0 software predicted many potential targets of TNK2‐AS1 and miR‐150‐5p was further selected for investigation, as miR‐150‐5p was reported to suppress cell proliferation and migration in various types of cancers.^22^, ^23^ In the cardiovascular research, miR‐150‐5p inhibited the endothelial proliferation, migration and tube formation.^24^ MiR‐150‐5p also exerted inhibitory effects on the proliferation and migration of pulmonary arterial smooth muscle cells under hypoxic conditions.^25^ On the other hand, in vivo animal studies miR‐150 knockout mice showed the reduced atherosclerosis lesion size and inflammatory response,^26^ and secreted monocytic miR‐150 enhanced endothelial cell migration.^27^ These discrepancies may require further investigations. Our data showed that TNK2‐AS1 repressed miR‐150‐5p expression and enforced expression of miR‐150‐5p restored the enhanced effects of TNK2‐AS1 on HASMC proliferation and migration, indicating TNK2‐AS1 regulated HASMC proliferation and migration via acting as a ceRNA for miR‐150‐5p. Recent evidence showed that miR‐150 is closed related to mitochondrial functions including glucose uptake, mitochondrial membrane potential and redox potential.^28^ Thus, the involvement of TNK2‐AS1/miR‐150 axis in regulating mitochondrial functions of HASMCs may require further studies.

MiRNAs exerted the functional actions via targeting the 3’UTR of the genes, which subsequently repressed the targeted genes. In order to find out the targeting genes of miR‐150‐5p, we used TargetScan tool to do the prediction. Among these predicted genes, we chose VEGFA and FGF1 for additional investigation. Previous studies have shown that VEGFA exerted enhanced effects on the VSMC proliferation as well as differentiation.^29^, ^30^, ^31^ In terms of FGF1, inhibition of FGF1 suppressed VSMC proliferation and migration, while overexpressed FGF1 in VSMCs exerted enhanced effects on cell proliferation and migration.^32^, ^33^, ^34^, ^35^, ^36^ In our study, miR‐150‐5p exerted suppressive effects on ox‐LDL‐induced hyper‐proliferation and migration of HASMCs, which were attenuated by enforced expression of VEGFA and FGF1. More importantly, TNK2‐AS1 knockdown effects on ox‐LDL‐stimulated HASMC proliferation and migration could be partially restored by the enforced expression of VEGFA and FGF1. Collectively, these data implied that the regulatory role of TNK2‐AS1 in ox‐LDL‐stimulated HASMCs may involve miR‐150‐5p/VEGFA/FGF1 signalling pathways.

To summarize, our results showed that TNK2‐AS1 was up‐regulated in the ox‐LDL‐stimulated HASMC model of atherosclerosis. Further findings showed that TNK2‐AS1 showed enhanced effects on HASMC proliferation and migration, and mechanistic studies implied that TNK2‐AS1 exerted its action in ox‐LDL‐stimulated HASMCs via regulating VEGFA and FGF1 expression by acting as a ceRNA for miR‐150‐5p. Our study suggested that targeting TNK2‐AS1/miR‐150‐5p/VEGFA/FGF1 axis may be effective in attenuating atherosclerosis.

## CONFLICT OF INTEREST

None.

## AUTHOR CONTRIBUTIONS

TC and MC researched conception and design. TC, XC and KZ performed the experiments and analysed data. AZ and BL interpreted the results. TC and MJ drafted the manuscript. Both authors read and approved the final manuscript.

## Data Availability

The data sets analysed during the current study are available from the corresponding author on reasonable request.
